# Biological Properties and the Role of IL-25 in Disease Pathogenesis

**DOI:** 10.1155/2018/6519465

**Published:** 2018-09-23

**Authors:** Yuwan Liu, Zewei Shao, Guoqiang Shangguan, Qingli Bie, Bin Zhang

**Affiliations:** ^1^Department of Laboratory Medicine, Affiliated Hospital of Jining Medical University, Jining, Shandong, China; ^2^Institute of Forensic Medicine and Laboratory Medicine, Jining Medical University, Jining, Shandong, China

## Abstract

The interleukin- (IL-) 17 superfamily, a T cell-derived cytokine, consists of 6 ligands (IL-17A–IL-17F) and 5 receptors (IL-17RA–IL-17RE). IL-17A, a prototype member of this family, is involved in the pathogenesis of allergies, autoimmune diseases, allograft transplantations, and malignancies. By contrast, IL-17B is reported to be closely related to certain diseases, particularly tumors such as breast cancer, gastric cancer, and pancreatic cancer. Recently, the biological function of IL-17E (also called IL-25) in disease, particularly airway diseases, has attracted the attention of researchers. However, studies on IL-25 are scant. In this review, we detail the structural characteristics, expression patterns, responder cells, biological properties, and role of IL-25 in disease pathogenesis.

## 1. Introduction

Cytokines are a class of small molecular proteins with broad biological activity. They are synthesized and secreted by immune cells (monocytes, macrophages, T cells, B cells, and natural killer (NK) cells) and nonimmune cells (endothelial cells, epidermal cells, and fibroblasts). Cytokines can regulate innate and adaptive immunities, blood-cell production, cell growth, adult pluripotent stem cells (APSC), pluripotent cells, and damaged tissue repair. The interleukin- (IL-) 17 family is a recently discovered group of cytokines that share homology in amino acid sequences and have highly conserved cysteine residues [1]. The IL-17 family and its receptors, which share minimal homology with other cytokines or known proteins, have been recognized as a distinct cytokine-receptor family and are crucial for normal host immune responses; this family is associated with many human pathogeneses, including those of inflammation and cancer [2–4].

## 2. Structural Characteristics of IL-25

IL-25, also named IL-17E, was first reported by Lee et al. [5] as a new member of the IL-17 family. Shortly after, Fort et al. reported that IL-25 is a cytokine produced by type 2 helper T (Th2) cells with structural similarity with IL-17 [6]. IL-25 was discovered after a BLAST search of the NCBI expression sequence tag (EST) database. A sequence of EST with a significant homology to IL-17 was discovered, and the IL-25 gene was finally cloned through reverse genetics. The IL-25 gene is located on chromosome 14 (14q11.2); it is 3987 base pairs (bp) in length and contains a 483 bp open reading frame, encoding a 161-amino acid hydrophobic signal peptide. The final products include a hydrophobic signal peptide consisting of 16 amino acids and a mature protein composed of 145 amino acids [7]. The IL-25 gene has two types of alternative splicing mRNA products that encode two subtypes (subtypes 1 and 2). The mRNA of both subtypes contains two exons; subtype 2 is less of an internal fragment than subtype 1 for a shorter N end. The mRNA of subtype 1 encodes a protein composed of 177 amino acids, and the mRNA of subtype 2 encodes a protein with 161 amino acids. Both subtypes have the same carboxy-terminal end composed of 159 amino acids. So far, no studies have reported differences in the physiological function of the two subtypes [8]. The murine IL-25 gene is located on chromosome 7, measures 985 bp in length, and encodes a protein composed of 169 amino acids. The human and mouse IL-25 genes share 80% homology. The IL-25 proteins of the human and mouse have a potential N-glycosylation site and a conserved cysteine sequence, which is composed of 10 cysteine residues in humans and 11 cysteine residues in mice [6].

## 3. Expression Patterns of IL-25

Fort et al. reported that IL-25 is a cytokine produced by Th2 cells, which are one of the earliest recognized sources of IL-25 [6]. Subsequently, the bone marrow-derived mast cells [9], alveolar epithelial cells [10], and alveolar macrophages were identified as other sources of IL-25 [11]. Later, the IL-25 expression was identified in the central nervous system [12] and the bronchial submucosa in asthmatic patients [13]. Sonobe et al. proved that IL-25 can be produced by brain capillary endothelial cells (BCECs) [14]. Gregory et al. reported the expression of IL-25 in varying degrees in allergic diseases [15]. A recent study has shown that mesenchymal stem cells derived from the placenta and bone marrow also secrete IL-25 [16]. A series of experimental studies have found that IL-25 is widely distributed and can be expressed in various cells, tissues, and systems.

## 4. IL-25 Responder Cells

The effects of IL-25 are mediated by the IL-25 receptors that are composed of two subunits, IL-17RA and IL-17RB [5]. Terashima et al. reported that NKT cells are target cells of IL-25 [17]. Stock et al. further proved that IL-17RB is highly expressed on a subset of inactive and activated CD4(+) invariant NKT (iNKT) cells [18]. Subsequently, type 2 myeloid cells, Th9 cells, basophils, eosinophils, mast cells, and endothelial cells [19] were identified as target cells of IL-25 in the course of allergic inflammation [20–22]. Recently, Yang et al. reported that macrophages carry IL-17RB [23], and Hongjia et al. proved that dendritic cells carry IL-17RB as well [24].

## 5. Biological Activities of IL-25

IL-25 can induce and enhance Th2-type immune responses and plays an important role in some allergic diseases. However, how IL-25 regulates the Th2 immune response is not fully understood. Some studies have suggested that IL-25 enhances the expression of Th2-type cytokines and induces Th2-type immune responses mainly through two mechanisms: (i) high doses of IL-25 can induce inherent lymphoid type 2 cells (ILC2s) to produce IL-4, IL-5, IL-13, and other cytokines and (ii) low doses of IL-25 can induce Th cells to differentiate into Th2 cells with the participation of cluster of differentiation 4 (CD4^+^) T cells and increase the expression of Th2-type cytokines [25, 26].

In addition to promoting Th2-type immune responses, IL-25 can also inhibit the immune responses mediated by Th1/Th17. Kleinschek et al. found that IL-25 in knockout mice were highly susceptible to autoimmune encephalomyelitis and rapid deterioration [12]. Through a study of patients with inflammatory bowel disease, Caruso et al. found that IL-25 can inhibit IL-12 production, reduce inflammation mediated by Th1, and inhibit Th17 immune responses by inducing IL-23 production [27].

## 6. Role of IL-25 in Asthma

**Figure 1:**
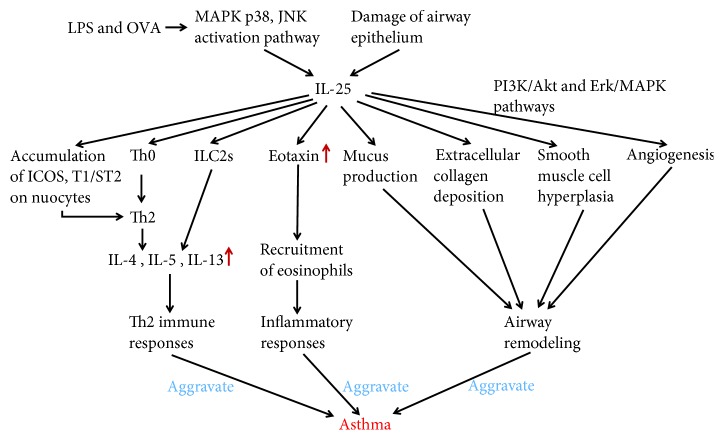
The potential mechanism of IL-25 in asthma. LPS and OVA induce the production of IL-25 in bronchial epithelial cells via activating MAPK p38 and JNK. Damage of airway epithelium induces the production of IL-25 in bronchial epithelial cells as well. IL-25 can enhance the Th2-type immune responses, stimulate ILC2s, promote the accumulation of inducible costimulator (ICOS) and T1/ST2 on nuocytes, or induce naïve T cells differentiated into Th2 cells to produce IL-4, IL-5, IL-13, or other cytokines. IL-25 increases chemokines and promotes the recruitment of eosinophils and inflammation. IL-25 can also promote airway remodeling by mediating mucus secretion, extracellular collagen deposition, smooth muscle cell proliferation, and angiogenesis. All of the above can aggravate asthma.

Bronchial asthma is a chronic inflammatory disease of the airways, which is caused by various cells (e.g., eosinophils, mast cells, T lymphocytes, neutrophils, smooth muscle cells, and airway epithelial cells) and cellular components. The pathogenesis of asthma is related to Th2 cells, ILC2s, Th2 cytokines secreted by Th2 cells and ILC2s, and epithelial cell factors [28]; however, the pathogenesis of asthma has not yet been fully clarified. IL-25 is associated with bronchospasm after aspirin challenge, possibly via mechanisms other than altered LTC4 and PGD2 production [29]. Blockade of the IL-25 receptor (IL-25R) reduced many rhinovirus-induced exacerbation-specific responses, including type 2 cytokine expression, mucus production, and recruitment of eosinophils, neutrophils, basophils, and T and non-T type 2 cells [30, 31]. The release of IL-25 has been found to increase when the airway epithelium has been damaged, and this plays an important role in allergic diseases represented by bronchial asthma [2, 32, 33]. Similarly, Wang et al. found that IL-25 promoted the accumulation of inducible costimulator (ICOS) and T1/ST2 on nuocytes, further inducing the proinflammatory Th2 cells, and promoted Th2 cytokine responses in ovalbumin-induced airway inflammation [34]. Eosinophils are considered a typical marker of bronchial asthma airway inflammation [35]. IL-25 through immune reactivity localize with eosinophils [19]. Wong et al. suggested that IL-25 can activate eosinophils in allergic inflammation, while levels of IL-4, IL-5, eosinophil chemokines, and IgE increased [36]. The IL-25/IL-25R axis plays a crucial role in promoting the recruitment and proinflammatory function of eosinophils in allergic asthma [37]. It also plays an important role in the recruitment of eosinophils, airway mucus oversecretion, and airway remodeling in the airway of mice [2, 6, 38, 39]. Corrigan et al. found that IL-25 contributes to angiogenesis, at least partly by increasing endothelial cell VEGF/VEGF receptor expression through PI3K/Akt and Erk/MAPK pathways [40]. IL-25 can also mediate bronchial smooth muscle hyperplasia and collagen deposition around the airway [15], which further supports the idea that IL-25 promotes airway remodeling. IL-25 and its receptor IL-17RB are considered as targets for innate and adaptive immune responses in chronic allergic airway disease [41]. Specific immunotherapy reduced asthmatic Th2 cytokine levels and the production of IL-25 and alleviated oxidative stress and cell apoptosis in the lung tissue of an asthma mouse model [42]. Lipopolysaccharide and ovalbumin (OVA) induced the production of IL-25 in bronchial epithelial cells in vitro via the activation of MAPK p38 and JNK [34]. Zhang et al. reported that the coblockade of IL-13 and IL-25 with sIL-13R and sIL-25R was more effective than either agent alone at decreasing inflammatory cell infiltration, airway hyperresponsiveness, and airway remodeling, including mucus production, extracellular collagen deposition, smooth muscle cell hyperplasia, and angiogenesis in mice exposed to OVA [43]. Bronchial mucosal vascular remodeling refers to structural changes such as loss of epithelial integrity. Chronic exposure of the airways to IL-25 alone is sufficient to cause functionally relevant airway remodeling, with the corollary that targeting of IL-25 may attenuate bronchial remodeling and fibrosis in human asthmatics [44]. To target IL-17Rb^+^CD4^+^NKT cells for the treatment of allergic asthma, IL-25 is considered to be a novel therapeutic approach [17]. In conclusion, IL-25 plays a key role in the pathogenesis of bronchial asthma, and the regulation of IL-25 production is expected to become a new direction for the treatment of bronchial asthma (Figure 1).

## 7. Role of IL-25 in Rheumatoid Arthritis

**Figure 2:**
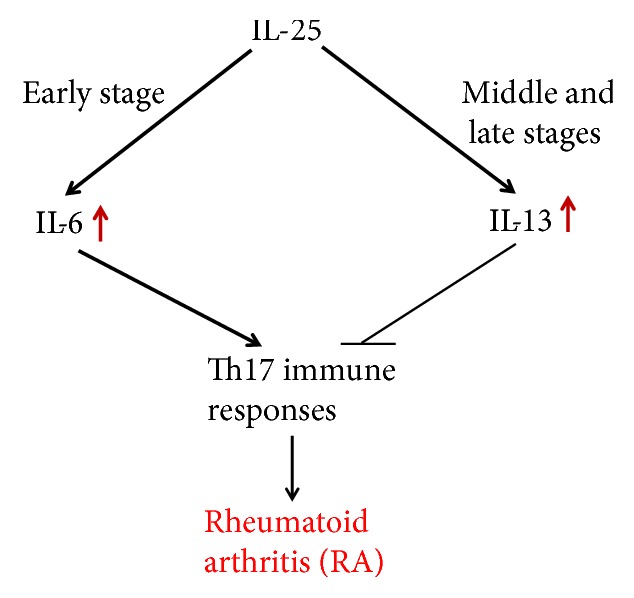
The mechanism of IL-25 involvement in rheumatoid arthritis. IL-25 stimulates the production of IL-6 at the early stage of RA to promote Th17 immune responses. IL-25 also suppresses Th17 immune responses in an IL-13-dependent manner at the middle and late stages of RA.

Rheumatoid arthritis (RA) is a chronic, systemic autoimmune disease characterized by erosive and symmetrical arthritis. The basic pathological changes of RA include synovitis, pannus formation, and gradual joint cartilage and bone destruction, which may eventually lead to joint malformation and loss of function. The exact pathogenesis of RA is unknown, but it is classified as an immune-mediated inflammatory disorder. Studies have shown that RA inflammation is dominated by Th1 cell immunity and that there is an imbalance of Th1 cell polarization. The inflammatory damage caused by this immune imbalance is closely related to Th17 cells and IL-17 family members [45]. Some researchers have found that the expression of IL-25 in articular cartilage inhibits the synthesis of articular cartilage matrix, stimulates the release of nitric oxide (NO), and stimulates the production of IL-6, which is related to the occurrence of arthritis. Recent studies [10, 27, 46] suggest that IL-25 has dual immune-regulatory effects: it can upregulate Th2-mediated immune responses and also downregulate Th1 and Th17 cell-mediated immune responses. Moreover, collagen-induced arthritis (CIA) in a mouse model showed high expression of IL-25 and IL-17 in the early stage of diseases [47]. In a recent study, Liu et al. found that IL-25 can alleviate CIA development in mice through suppression of Th17-type immune responses in an IL-13-dependent manner [48]. In conclusion, IL-25 may be involved in the immune and inflammatory responses of RA and has considerable value in the treatment of RA (Figure 2).

## 8. Role of IL-25 in Allergic Rhinitis

Allergic rhinitis (AR) is considered a nasal inflammation mediated by Th2-type cytokines, characterized by the aggregation of nasal mucosal eosinophils and mast cell and increased serum antigen-specific IgE levels [49]. It is a common and recurrent disease in the ear-nose-throat department that severely affects the quality of life of the patients. In recent years, the imbalance of Th1/Th2 cytokines has been discovered to play an important role in the pathogenesis of AR: Th2 cytokines increase while Th1 cytokines decrease, breaking the balance between them, which is the basis of AR. The pathogenesis of AR and bronchial asthma is very similar; both are Th2-type immune hyperactivity reactions and diseases essentially caused by Th2-type hyperimmune reaction. Grossman [50] introduced the concept of “the same airway, the same disease.” Casale and Dykewicz have found that AR and bronchial asthma are highly similar in their etiology, pathogenesis, and treatment, further proving this concept [51]. Hence, we speculate that the role of IL-25 in bronchial asthma patients is also applicable in the ARAs and that eosinophils are associated with allergic diseases, such as AR and bronchial asthma [52]. IL-25 can promote the expression of Th2 cytokines and accumulation of eosinophils [6, 26], inhibit the apoptosis of eosinophils, enhance adhesion between eosinophils and epithelial cells, release cytokines and chemokines by stimulating eosinophils, and thus promote allergy [2, 6, 36, 38, 39, 45, 53, 54]. These studies have confirmed that IL-25 is involved in the pathogenesis of allergic rhinitis and that it plays a crucial role in the occurrence and development of the disease. The expression of IL-25 in the nasal mucosa and the concentration of IL-25 in the serum are positively correlated with the severity of AR, which can be used to judge the severity of allergic rhinitis. Therefore, inhibitors related to IL-25 may be a new target for the treatment of allergic rhinitis.

## 9. Role of IL-25 in Inflammatory Bowel Disease

**Figure 3:**
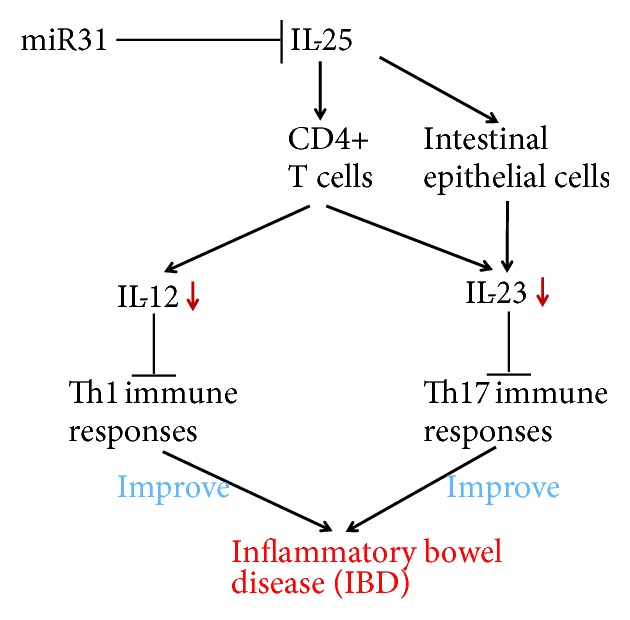
The potential mechanism of IL-25 in IBD. IL-25 stimulates CD4^+^ T cells to reduce synthesis of IL-12 and inhibit Th1 immune responses. IL-25 also stimulates CD4^+^ T cells and intestinal epithelial cells to decrease IL-23 to inhibit Th17 immune responses, thus improving inflammatory bowel diseases.

Inflammatory bowel disease (IBD) is an idiopathic intestinal inflammatory disease involving the ileum, rectum, and/or colon. The clinical manifestations are diarrhea, abdominal pain, and even persistent loose stools. The most common forms of the disease are ulcerative colitis (UC) and Crohn's disease (CD). The etiology and pathogenesis of IBD are not completely clear, and recent studies suggest that IBD is caused by the interaction of several factors, for example, environmental, genetic, and immune factors. Its pathogenesis is related to the regulation of the intestinal mucosal immune barrier to the inflammatory response of the intestinal antigen, and Th1/Th17-type reaction mediated by IL-12/23 is one of the key factors [55]. Commensal-dependent expression of IL-25 by intestinal epithelial cells limits the expansion of Th17 cells in the intestine by inhibiting expression of macrophage-derived IL-23 [46]. Kleinschek et al. found that IL-25 knockout mice are highly susceptible to autoimmune encephalomyelitis, which was associated with increased expression of IL-23; IL-17, interferon *γ*, tumor necrosis factor *α*, and other proinflammatory factors infiltrate the central nervous system [12]. Caruso et al. used IL-25 to stimulate CD4^+^ cells in the intestinal mucosal tissue of CD patients, which lead to decreased synthesis of IL-23 and IL-12, similar to the levels in the peripheral blood [27]. Su et al. found that the level of IL-25 in the intestinal mucosa and serum of patients with active IBD was significantly lower than that of the control group and was negatively correlated with the degree of IBD activity and the level of C-reactive protein [56]. A recent study by Shi et al. showed that the expression of IL-25 was negatively correlated with microRNA-31 in rats with CD and patients with UC. Luciferase test results showed that miR-31 could bind to the untranslated region of IL-25 mRNA 3′ and directly regulate the expression of IL-25. The content changes of microRNA-31 in CD rats can affect the Th1/Th17 pathway mediated by IL-12/23 in the intestinal mucosa and consequently improve or aggravate colitis [57]. If IL-25 in CD mice was cleared or the content of IL-25 in the colon was decreased, the treatment effects of miR-31 inhibitors on colitis were significantly decreased. The results suggest that IL-25 is an important anti-inflammatory factor in the pathogenesis of IBD and a possible target to inhibit the Th1/Th17 inflammatory pathways, which are mediated by IL-12/IL-23. In the future, IL-25 inhibitors may be a new therapy for the treatment of IBD with potential quality of life benefits for patients with IBD (Figure 3).

## 10. Role of IL-25 in Skin Diseases

Urticaria is a localized edema caused by a temporary increase in vascular permeability of the skin and mucous membrane. Chronic urticaria (CU) is defined as skin lesions that recur for more than 6 weeks with attacks occurring at least 2 times per week. The pathogenesis of this disease is not clear. However, several studies have demonstrated that the imbalance of Th1/Th2 and Th2-mediated immune response is dominant in the pathogenesis of CU [58–60]. As explained earlier, IL-25 can increase Th2 cytokines via two mechanisms, resulting in enhanced Th2-type immune response. Therefore, IL-25 may be involved in the pathogenesis of CU.

**Figure 4:**
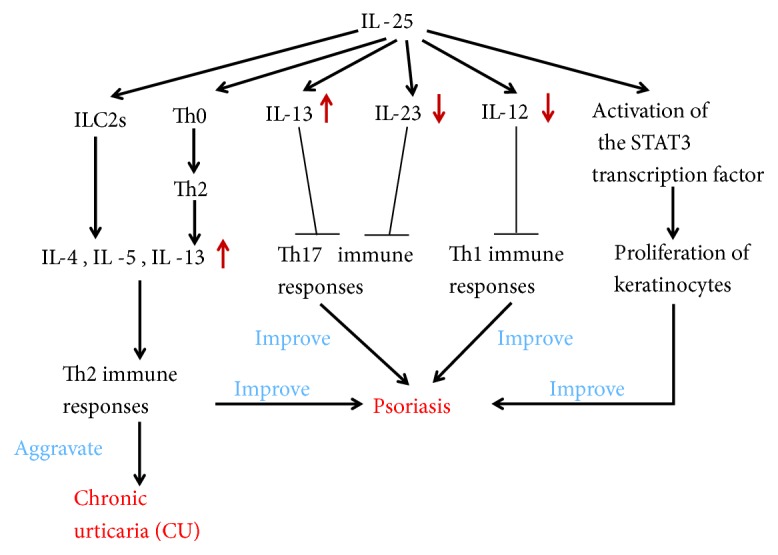
The role of IL-25 in skin diseases. IL-25 can aggravate CU by enhancing the Th2-type immune responses. IL-25 stimulates the proliferation of keratinocytes via activation of the STAT3 transcription factor, inhibits the production of IL-12 to reduce Th1 immune responses, increases the production of IL-13, and decreases the production of IL-23 to inhibit the Th17 immune responses. Thus, IL-25 can improve psoriasis.

Psoriasis is a polygenic inflammatory dermatosis that can be induced by certain environmental factors [61]. It is typically characterized by scaly erythema or plaque that can be limited or widely distributed. The exact etiology and pathogenesis of psoriasis are not yet clear, but a series of studies have shown that Th1/Th2 imbalance and Th17 cells comediate this autoimmune disease [62, 63]. IL-25 is well known to regulate allergic responses and type 2 immunity. Caruso et al. found that IL-25 levels in the peripheral blood of patients with psoriasis vulgaris were significantly reduced as compared to normal people [27]. Thus, IL-25 may play a certain inhibitory role in the pathogenesis of psoriasis vulgaris. Recently, Xu et al. have shown that via IL-17RB expression in keratinocytes, IL-25 stimulated the proliferation of keratinocytes and induced the production of inflammatory cytokines and chemokines, via activation of the STAT3 transcription factor [64]. Thus, the IL-17-induced autoregulatory circuit in keratinocytes is proved to be mediated by IL-25, and this circuit could be targeted in the treatment of psoriasis patients (Figure 4).

## 11. Relationship between IL-25 and Other Diseases

Bernal et al. found that the expression of IL-25 in the peripheral blood of patients with nonalcoholic fatty liver disease (NAFLD) is significantly decreased and is negatively correlated with body mass index (BMI) [65]. In vitro and in vivo studies have further confirmed that IL-25 can activate macrophages, transform macrophages into M2 type, enhance its intake of fat, promote fat decomposition, inhibit fat synthesis, and significantly improve NAFLD. Moreover, fulminant hepatitis (FH) is a liver disease characterized by massive destruction of hepatocytes and severe impairment of liver functions [65]. In a FH animal model study, Sarra et al. found that the IL-25 content in the liver of FH mice was significantly reduced and the intervention of IL-25 before drug induction could prevent the occurrence of FH, suggesting that IL-25 might have therapeutic effects on FH [66].

**Figure 5:**
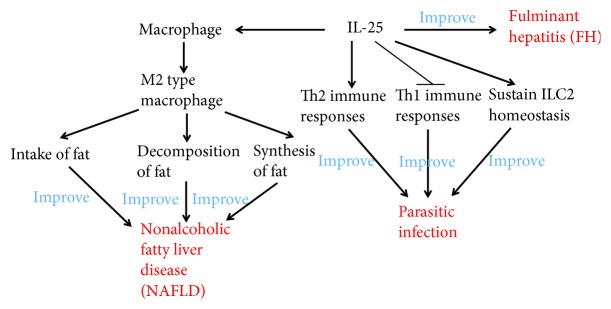
The relationship between IL-25 and other diseases. IL-25 can activate macrophages, transform macrophages into M2 type, enhance its intake of fat, promote fat decomposition, inhibit fat synthesis, and significantly improve NAFLD. IL-25 enhances Th2 immune responses, inhibits Th1 immune responses, sustains ILC2 homeostasis, and consequently improves parasitic infection. IL-25 is associated with FH as well.

Owyang et al. found that IL-25 not only enhanced Th2 immune response but also inhibited the secretion of Th1-type cytokines to inhibit gastrointestinal inflammation induced by parasite in a mouse model of whipworm infection [67]. Fallon et al. found that IL-25 knockout mice had delayed secretion of Th2 cytokines and could not effectively expel the nematode, further confirming that IL-25 played an important role in parasitic infection [68]. Parasitic helminths and allergens induce a type 2 immune response leading to profound changes in tissue physiology, including hyperplasia of mucus-secreting goblet cells and smooth muscle cell's hypercontractility. Tuft cells express IL-25, sustain ILC2 homeostasis, and regulate type 2 immune responses in mice [69, 70] (Figure 5).

## 12. Conclusion

As one of the members of the IL-17 family, IL-25 is distinctly different from other family members in molecular structure and biological functions. The current studies show that IL-25 not only plays an important role in the regulation of type 2 immune responses and inflammatory, skin, and autoimmune diseases but also has a certain role in the treatment of tumors. Therefore, IL-25 provides new exploratory direction and is expected to become a new treatment target for these diseases. However, further research is needed to confirm these results.
